# PALB2 mutations in breast cancer patients from a multi-ethnic region in northwest China

**DOI:** 10.1186/s40001-015-0182-9

**Published:** 2015-10-21

**Authors:** Yong Tao Li, Wei Hua Jiang, Xiao Wen Wang, Ming Shuai Zhang, Cheng Guang Zhang, Li Na Yi, Fulati WuwaliKhan, Aisikaer Ayoufu, Jiang Hua Ou

**Affiliations:** Department of Breast Surgery, Cancer Hospital, Xinjiang Medical University, Urumqi, China; Cancer Hospital, Xinjiang Medical University, 789 Eeast Suzhou Street, Urumqi, 830011 China

**Keywords:** PALB2, Breast cancer, Genetic testing, Mutation

## Abstract

**Background:**

Germline mutations in PALB2 gene make a small contribution to heritable breast cancer susceptibility. A recent report has revealed that women with mutations in the PALB2 gene were more than nine times as likely to develop breast cancer compared to those without. The aim of this study is to understand the status of PALB2 mutations among Chinese high-risk breast cancer patients in a multi-ethnic region in China.

**Methods:**

152 patients with hereditary predisposition to breast cancer from the Xinjing region of China were enrolled in the study, and 100 control samples from healthy women were collected in the same locality. We sequenced the coding sequences and flanking intronic regions of PALB2 gene from DNA samples obtained from all subjects by direct sequencing.

**Results:**

A total of 4 deleterious PALB2 mutations were identified in 152 breast cancer patients with a prevalence of about 2.6 % (4/152). The PALB2 mutation prevalence was 3.2 % (3/95) in cases with family history of breast cancer. In addition to the four deleterious mutations, we identified nine missense variants in 12 patients, using the prediction Softwares SIFT and PolyPhen, four of which might be disease associated (in 5 patients). Two of the 4 patients with deleterious mutations and 2 of the 5 patients presenting putative deleterious missense mutations had triple-negative breast cancer. No PALB2 mutation carriers were identified in 100 healthy controls.

**Conclusion:**

PALB2 mutations account for a small, but not negligible, proportion of patients with hereditary predisposition to breast cancer in the Xinjing region of China.

## Background

Breast cancer is one of the most common cancers in women worldwide [1]. However, the exact mechanisms of breast cancer are still poorly understood. Previous studies showed that breast cancer has an inherited component [2], and the high-penetrance breast cancer predisposing genes, BRCA1 and BRCA2, account for up to 10–40 % of inherited breast cancer [3, 4]. Other relevant genes also contribute to inherited breast cancer, among these genes is PALB2 (Partner and Localizer of BRCA2) gene. PALB2 is located on chromosome 16p12.2 spanning approximately 38 kb, containing 13 exons and 12 introns, and encodes for a protein involved in BRCA2-related pathways [5]. PALB2 indirectly affects the expression of BRCA2, and the loss-of-function mutations in BRCA2 usually cause genetic instability, avoiding the defense system, resulting in non-controlling cell proliferation and thereby inevitably leading to tumorigenesis [6, 7]. It has been discovered that heterozygosity for loss-of-function mutations at PALB2 increases risk of developing breast cancer two- to ninefold [8, 9].

Inherited PALB2 mutations associated with increased risks of developing breast cancer have been identified in families from many parts of the world, including China. To date, only a few studies have been reported on PALB2-associated breast cancer in China, and the revaluated studies only involved ethnic Han Chinese [10, 11]. China is a multiple ethnic country comprising 56 ethnic groups. Xinjiang is the largest administrative region located in the northwest of China. It is home to a number of different ethnic groups including the Han, Uyghur, Kazakh, Hui, Kyrgyz, Mongol, etc. But thus far, inherited PALB2 mutations has not been screened in Xinjiang population. For this purpose, we sequenced the complete coding and flanking regulatory regions of PALB2 from constitutional DNA of 152 patients with hereditary predisposition to breast cancer, all previously employed to study the distribution of germline mutations in BRCA1, BRCA2 [12, 13]. Here, we present the identification of PALB2 mutations in patients with inherited breast cancer from Xinjiang region of China.

## Methods

### Subjects

All patient cases were recruited from Xinjiang, China. They had a definite pathological diagnosis of breast cancer and received the standard treatment in our hospital during the period 2005–2014. Participants were patients with breast cancer who had family history, early onset, male breast cancer, or bilateral breast cancer (BI-BC). A total of 152 patients were eligible for testing. Among them, 33 were early onset cases diagnosed below 35 years of age, 95 cases had family history of breast cancer, 24 had bilateral breast cancer, 4 had male breast cancer, and 48 had triple-negative breast cancer. In the included patients, the ethnic composition were 104 Han, 28 Uyghur, 8 Hui, 7 Kazakh, 3 Mongol, and 2 Xibo. The mean age at diagnosis was 40.6 years (range 24–73 years). DNAs from 100 control samples from healthy women were collected in the same locality. The control group from the health women of the outpatient clinic had no family history of cancer. Their blood was collected and they agreed to give blood. The age of the individuals was range 20–62 years.

The study was approved by the Ethics Committee (IRB approval number: XJYD1320) of the Cancer Hospital of Xinjiang Medical University, and informed consent was obtained from each participant.

### Genomic DNA sequencing

Genomic DNA was isolated from 5 ml of peripheral blood and stored in 10 mM Tris (pH8) EDTA at 4 °C. PALB2 exons, flanking intronic regions (50–100 bp in length), and 5′-and 3′-untranslated regions (UTR) were evaluated from all subjects. We adopted the direct sequencing method. DNAs were amplified using Platinum Taq DNA polymerase (Invitrogen). Sequence reactions were used on the Illumina HiSeq and MiSeq platforms. Sequences were analyzed using ANNOVAR software.

### Prediction of pathogenicity of missense variants

Potential consequences of missense mutations were obtained using the prediction Softwares SIFT (http://sift.jcvi.org/) and PolyPhen (http://genetics.bwh.harvard.ed-u/pph).

## Results

### PALB2 deleterious mutations

PALB2 deleterious mutations were identified in 4 patients (Table 1) including a novel frameshift mutation in exon 4(c.1039_1040insA,p.E347fs) and exon 12(c.3294_3298delTCGTC,p.1098_1100del) in addition to a frameshift mutation in exon 4(c.509-510delGA,p. R170fs) and a truncating mutation in exon 5(c.2386G>T,p.G796X) that have been previously reported [14] in HGMD (Human Gene Mutation Database). The prevalence of the PALB2 germline mutation was about 2.6 % (4/152). No PALB2 mutation carriers were identified in 100 healthy controls. In the 152 patients, 95 cases had family history of breast cancer, three patients were identified deleterious mutations, the PALB2 mutation prevalence was 3.2 % (3/95).

**Table 1:** Inherited truncating mutations in PALB2

Genomic locale	Exon	Nucleotide change	Protein change	Type
chrl6:23,619,237-41	12	c.3294_3298delTCGTC	p.1098_1100del	Frameshift
chrl6:23,646,828	4	c.1039_1040insA	p.E347fs	Frameshift
chrl6:23,647,357-58	4	c.509-510delGA	p. R170fs	Frameshift
chrl6:23,641,089	5	c.2386 G>T	p.G796X	Nonsense

### PALB2 missense variants and prediction of pathogenicity

In addition to the four deleterious mutations, we identified nine missense variants in 12 patients (Table 2) including 8 novel missense variants in exon 4(c.1273G>A,p.V425M; c.925A>G, p.I309V; c.691A>G,p.K231E), exon 10(c.3035C>T,p.T1012I), exon 12(c.3306C>G,p.S1102R), exon 7(c.2720A>G,p.E907G; c.2699C>T,p.A900V) and exon 2(c.64G>A,p.A22T). The missense variants in exon 4(c.925A>G,p.I309V) was also identified in one patient of 100 healthy controls. To determine whether the missense variants were pathogenic, we further analyzed them using the prediction Softwares SIFT and PolyPhen. The application of the two different prediction programs and conservation profiles indicated that only four of them might be disease associated (c.3035C>T,p.T1012I; c.3054G>C,p.E1018D; c.2720A>G,p.E907G; c.64G>A,p.A22T).

**Table 2:** PALB2 missense variants and prediction of pathogenicity

Exon	Nucleotide change	Protein change	Mutation cases	SIFT score	Polyphen score
10	c.3035C>T	p.T1012I	1	0	0.997
7	c.2720A>G	p.E907G	1	0.02	0.983
4	c.1273G>A	p.V425M	1	0.01	0.002
4	c.925A>G	p.I309V	3	1	0
4	c.691A>G	p.K231E	1	0.04	0.023
10	c.3054G>C	p.E1018D	2	0.03	0.95
12	c.3306C>G	p.S1102R	1	0.01	0.119
7	c.2699C>T	p.A900V	1	0.02	0.024
2	c.64G>A	p.A22T	1	0	0.98

### Patients’ characteristics of PALB2 truncating and putative deleterious missense mutations

In total, we identified four deleterious mutations and four putative pathogenic missense mutations in the 152 cases. The family history and histopathology of the tumor tissues of the patients are indicated in Table 3. Among the four patients with deleterious mutations, 3 had family history of breast cancer and 1 had bilateral breast cancer, and 2 had triple-negative breast cancer (estrogen and progesterone receptor negative, and HER2/neu negative). Of the five patients with putative deleterious missense mutations, 4 had positive family history and 1 was an early-onset breast cancer. The patient with the PALB2 mutation, c.64G>A, was diagnosed with bilateral breast cancer and had positive family history.

**Table 3:** Patient’s characteristics of PALB2 truncating and putative deleterious missense mutations

Nucleotide change	Type	Ethnic group	Age at diagnosis	HR	HER-2	Inclusion reason
c.3294_3298delTCGTC	Frameshift	Han	65	1	1	2 daughters BC
c.509-510delGA	Frameshift	han	44	1	0	Mother and sister BC
c.1039_1040insA	Frameshift	Han	46	0	0	Bilateral BC
c.2386 G>T	Nonsense	Uyghur	33	0	0	mother BC
c.3035C>T	Missense	Han	45	1	0	sister BC
c.2720A>G	Missense	Kazakh	41	0	0	sister BC
c.3054G>C	Missense	Han	50	0	1	aunt BC
		Han	34	1	0	Early onset
c.64G>A	Missense	Han	50	0	0	BI-BC and mother BC

*BC* breast cancer, *BI-BC* bilateral breast cancer, *HR* hormone receptor, *HER-2* human epidermal growth factor receptor 2

In the included patients, 48 had triple-negative breast cancer, the PALB2 mutation prevalence was 4.2 % (2/48). Among the triple-negative breast cancers, 22 were selected cases with a family history or bilateral breast cancers, the PALB2 mutation prevalence was 9.1 % (2/22), for 2 of four patients with deleterious mutations had triple-negative breast cancer, and had family history or bilateral breast cancer.

## Discussion

In a recent report in the new England Journal of Medicine [9], women with inherited loss-of-function mutations in the PALB2 gene were found to be more than nine times as likely to develop breast cancer, compared to the general population. PALB2 was identified via a search for novel components of endogenous BRCA2-containing complexes and is critical for its localization to chromatin and recruitment to double-strand breaks [15]. PALB2 is also recruited by BRCA1 in response to DNA damage and serves as a linker between BRCA1 and BRCA2 necessary for BRCA2-mediated HR repair [16]. The PALB2 germline mutations and their carrier frequencies are 0.1–3.6 % among different populations [10, 14, 17–20], including China. Germline mutations in PALB2 gene make a small contribution to the heritable breast cancer susceptibility and increased risk for breast cancer.

The prevalence of PALB2 mutations varies due to founder mutation effects and other environmental and geographical factors. To date, only a very limited number of studies have focused on the association between PALB2 mutations and hereditary predisposition to breast cancer in China, and ethnic Han Chinese patients were enrolled in most of these studies, with no study addressing multiple ethnic groups in China. In our study, 152 specimen of patients with hereditary predisposition to breast cancer (including patients with early-onset breast cancer, family history of breast or ovarian cancer, bilateral cancer, or male breast cancer) were studied for PALB2 mutations. Four (2.6 %) of the patients were identified to harbor a deleterious PALB2 mutation. The result are consistent with the previous reports [10, 14, 17–20]. In the included 152 patients, the ethnic composition were 104 Han, 28 Uyghur, 8 Hui, 7 Kazakh, 3 Mongol, and 2 Xibo. Among the four patients with deleterious mutations, 3 were Han, 1 was Uyghur, so variation of the PALB2 mutations among different ethnic groups could not be obtained, for the number of participants in some of the groups was not sufficient for statistical analysis and comparison.

Of the 4 PALB2 deleterious mutations identified in our study, 2 have been previously reported and 2 were novel (Table 1). An interesting mutation of c.509–510delGA carried by ethnic Han Chinese patient was first described (with the highest frequency so far) in the Polish population [21]. Other studies reporting this mutation were all carried by European ancestry on their familial lineages with histories of breast cancer, suggesting that this mutation may have a European origin. PALB2 c.2386G>T has also been reported in families from Europe [22], with much lower frequency or in single patients. The mutation was carried by ethnic Uyghur Chinese patient in our study. PALB2 c.509-510delGA and c.2386G>T has been first reported in Asia; Is it a founder mutation? The spectrum of PALB2 mutations in the Chinese population may be larger if larger groups are analyzed. In this study, we identified nine missense variants in 12 patients (Table 2). Using the prediction Softwares SIFT and PolyPhen, four of the missense variants (c.3035C>T,p.T1012I; c.3054G>C,p.E1018D; c.2720A>G,p.E907G; c.64G>A,p.A22T) indicated putative pathogenic mutation. Similar to the mutation spectrum observed in BRCA1/2, protein truncating mutations in PALB2 are associated with breast cancer. There is no evidence that missense mutations in PALB2 play a significant role in breast cancer predisposition [23, 24]. Further research is needed to clarify effects of these putative pathogenic missense mutations on protein function.

Male breast cancer is significantly associated with BRCA2 mutations [25]. Our previous study has shown that male breast cancer mutations were BRCA2 mutations.

A small number of the pedigrees reported in the literature to carry protein truncating PALB2 mutations included cases of male breast cancer [26]. In our study, the four cases of male breast cancer had no detectable PALB2 mutations. Truncating PALB2 heterozygous mutations have been identified in bilateral breast cancer patients [18]. In this study, one of the four patients with deleterious mutations was bilateral breast cancer. So we think that PALB2 mutations contribute to a small fraction of bilateral breast cancer in the Xinjiang region of China. Some information about the general morphology of breast tumors with PALB2 mutation is available. Carriers with a family history of breast cancer were more likely to have triple-negative breast cancer [27]. In our study, two of the four patients with deleterious mutations had also triple-negative breast cancer, and two of the five putative deleterious missense mutations were triple-negative breast cancers. As previously observed [13], triple-negative breast cancers tend to harbor BRCA1/2 mutation which appears to be slightly overrepresented in PALB2-related breast cancers. Therefore, PALB2 mutation might be associated with triple-negative breast cancer.

## Conclusion

The results show that mutations in PALB2 are rare, but along with BRCA1 and BRCA2 are key breast cancer susceptibility genes in the Xinjiang region of China. PALB2 mutation may be associated with triple-negative breast cancer, the same as immunohistochemical features of BRCA1/2 mutation-associated breast tumors.

## Abbreviations

PALB2: partner and localizer of BRCA2
BC: breast cancer
BI-BC: bilateral breast cancer
HR: hormone receptor
HER-2: human epidermal growth factor receptor 2

## References

[1] Benson JR, Jatoi I (2012). The global breast cancer burden. Future Oncol.

[2] Stephens PJ, Tarpey PS, Davies H, Van Loo P, Greenman C, Wedge DC (2012). The landscape of cancer genes and mutational processes in breast cancer. Nature.

[3] Turnbull C, Rahman N (2008). Genetic predisposition to breast cancer: past, present, and future. Annu Rev Genomics Hum Genet.

[4] Kurian AW (2010). BRCA1 and BRCA2 mutations across race and ethnicity: distribution and clinical implications. Curr Opin Obstet Gynecol.

[5] Tischkowitz M, Xia B (2010). PALB2/FANCN: recombining Cancer and Fanconi anemia. Cancer Res.

[6] Frankenberg-Schwager M, Gregus A (2012). Chromosoma instability induced by mammography X-rays in primary human fibroblasts from BRCA1 and BRCA2 mutation carriers. Int J Radiat Biol.

[7] Pauty J, Rodrigue A, Couturier A, Buisson R, Masson JY (2014). Exploring the roles of PALB2 at the crossroads of DNA repair and cancer. Biochem J.

[8] Hollestelle A, Wasielewski M, Martens JW, Schutte M (2010). Discovering moderate-risk breast cancer susceptibility genes. Curr Opin Genet Dev.

[9] Antoniou AC, Casadei S, Heikkinen T, Barrowdale D, Pylkäs K, Roberts J (2014). Breast-cancer risk in families with mutations in PALB2. N Engl J Med.

[10] Cao AY, Huang J, Hu Z, Li WF, Ma ZL, Tang LL (2009). The prevalence of PALB2 germline mutations in BRCA1/BRCA2 negative Chinese women with early onset breast cancer or affected relatives. Breast Cancer Res Treat.

[11] Chen P, Liang J, Wang Z, Zhou X, Chen L, Li M (2008). Association of common PALB2 polymorphisms with breast cancer risk: a case-control study. Clin Cancer Res.

[12] Jianghua Ou, Tao Wu, Sijmons Rolf, Ni Duo, Wenting Xu, Upur Halmurat (2013). Prevalence of BRCA1 and BRCA2 germline mutations in breast cancer women of multiple ethnic region in northwest China. J Breast Cancer.

[13] Li YT, Ni D, Yang L, Zhao Q, Ou JH (2014). The prevalence of BRCA1/2 mutations of triple-negative breast cancer in Xinjing multiple ethnic region of China. Eur J Med Res.

[14] Casadei S, Norquist BM, Walsh T, Stray S, Mandell JB, Lee MK (2011). Contribution of inherited mutations in the BRCA2- interacting protein PALB2 to familial breast cancer. Cancer Res.

[15] Sy SM, Huen MS, Chen J (2009). PALB2 is an integral component of the BRCA complex required for homologous recombination repair. Proc Natl Acad Sci USA.

[16] Zhang F, Ma J, Wu J, Ye L, Cai H, Xia B (2009). PALB2 links BRCA1 and BRCA2 in the DNA-damage response. Curr Biol.

[17] Wong MW, Nordfors C, Mossman D, Pecenpetelovska G, Avery-Kiejda KA, Talseth-Palmer B (2011). BRIP1, PALB2, and RAD51C mutation analysis reveals their relative importance as genetic susceptibility factors for breast cancer. Breast Cancer Res Treat.

[18] Bogdanova N, Sokolenko AP, Iyevleva AG, Abysheva SN, Blaut M, Bremer M (2011). PALB2 mutations in German and Russian patients with bilateral breast cancer. Breast Cancer Res Treat.

[19] Peterlongo P, Catucci I, Pasquini G, Verderio P, Peissel B, Barile M (2011). PALB2 germline mutations in familial breast cancer cases with personal and family history of pancreatic cancer. Breast Cancer Res Treat.

[20] Casadei S, Norquist BM, Walsh T, Stray S, Mandell JB, Lee MK (2011). Contribution of inherited mutations in the BRCA2 interacting protein PALB2 to familial breast cancer. Cancer Res.

[21] Dansonka-Mieszkowska A, Kluska A, Moes J, Dabrowska M, Now-Akowska D, Niwinska A (2010). A novel germline PALB2 deletion in Polish breast and ovarian cancer patients. BMC Med Gene.

[22] Casadei Silvia, Norquist Barbara M, Walsh Tom, Stray Sunday, Mandell Jessica B, Lee Ming K (2011). Contribution of Inherited Mutations in the BRCA2-Interacting Protein PALB2 to Familial Breast Cancer. Cancer Res.

[23] Teo ZL, Park DJ, Provenzano E, Chatfield CA, Odefrey FA, Nguyen-Dumont T (2013). Prevalence of PALB2 mutations in Australasian multiple-case breast cancer families. Breast Cancer Res.

[24] Tischkowitz M, Capanu M, Sabbaghian N, Li L, Liang X, Vallée MP (2012). Rare germline mutations in PALB2 and breast cancer risk: a population-based study. Hum Mutat.

[25] Wooster R, Bignell G, Lancaster J, Swift S, Seal S, Mangion J (1995). Identifcation of the breast cancer susceptibility gene BRCA2. Nature.

[26] Sauty de Chalon A, Teo Z, Park DJ, Odefrey FA, Hopper JL, kConFab (2010). Are PALB2 mutations associated with increased risk of male breast cancer?. Breast Cancer Res Treat.

[27] Heikkinen T, Kärkkäinen H, Aaltonen K, Milne RL, Heikkilä P, Aittomäki K (2009). The breast cancer susceptibility mutation PALB2 1592delT is associated with an aggressive tumor phenotype. Clin Cancer Res.

